# Brochosome-Inspired Metal-Containing Particles as Biomimetic Building Blocks for Nanoplasmonics: Conceptual Generalizations

**DOI:** 10.3390/biomimetics6040069

**Published:** 2021-12-10

**Authors:** Zoran Jakšić, Marko Obradov, Olga Jakšić

**Affiliations:** Department of Microelectronic Technologies, Institute of Chemistry, Technology and Metallurgy—National Institute of the Republic of Serbia, University of Belgrade, Belgrade 11000, Serbia; marko.obradov@nanosys.ihtm.bg.ac.rs (M.O.); olga@nanosys.ihtm.bg.ac.rs (O.J.)

**Keywords:** nanoplasmonics, submicrometer particles, brochosomes, antireflective structures, leafhoppers, metamaterials, bio-inspired designs

## Abstract

Recently, biological nanostructures became an important source of inspiration for plasmonics, with many described implementations and proposed applications. Among them are brochosome-inspired plasmonic microstructures—roughly spherical core-shell particles with submicrometer diameters and with indented surfaces. Our intention was to start from the nanoplasmonic point of view and to systematically classify possible alternative forms of brochosome-inspired metal-containing particles producible by the state-of-the-art nanofabrication. A wealth of novel structures arises from this systematization of bioinspired metal-containing nanocomposites. Besides various surface nanoapertures, we consider structures closely related to them in electromagnetic sense like surface nano-protrusions, shell reliefs obtained by nano-sculpting, and various combinations of these. This approach helped us build a new design toolbox for brochosome-inspired structures. Additionally, we used the finite elements method to simulate the optical properties of simple brochosome-inspired structures. We encountered a plethora of advantageous optical traits, including enhanced absorption, antireflective properties, and metamaterial behavior (effective refractive index close to zero or negative). We conclude that the presented approach offers a wealth of traits useful for practical applications. The described research represents our attempt to outline a possible roadmap for further development of bioinspired nanoplasmonic particles and to offer a source of ideas and directions for future research.

## 1. Introduction

The scientific and practical importance and impact of (nano)plasmonics cannot be overestimated [1,2,3,4,5,6]. Until today, it has already ensured a vast number of novel applications, many of them seemingly counterintuitive, controversial, and even impossible at a first glance. Some of these include optical elements with deeply subwavelength resolutions vastly exceeding Abbe’s limit [4]; novel generation of all-optical integrated circuits with optical operating frequencies and packaging densities of electronic integrated circuits [3], 2D resonant cavities vastly smaller than the operating wavelength [7]; chemical sensors that can sense minuscule amounts of analyte, even reaching the ultimate limit—the sensing of a single molecule [8]; plasmonic metamaterials with arbitrarily tailorable refractive index, including very high values [9], values near zero [10] and negative ones [11,12], etc. A whole new world of novel physics, chemistry, and biomedicine and their applications has opened with the advance of plasmonics. All of this stems from the fundamental capability of nanoplasmonics to arbitrarily transform the electromagnetic fields and to ensure extreme concentrations of fields at deep subwavelength level.

Nanoplasmonics uses nanocomposites that contain electric conductors based on free electrons (the plasmonic materials, among which most often used are noble metals). Their combination with bio-inspired designs might not seem a reasonable idea at a first glance. The reality has showed us that the situation is quite the opposite. Bringing together two seemingly incompatible paradigms, nanoplasmonics and biomimetics, not only has proven itself fruitful, but actually brought to a vast number of new results and is now considered one of the major directions of research in nanoplasmonics. In recent years, the field literally exploded with a vast number of novel applications [13,14,15,16,17,18,19,20,21,22,23,24,25,26]. Bringing complexity of organic tissues into the world of evanescent electromagnetic fields opened many new and unexpected pathways and ensured an almost inexhaustible and immensely powerful design toolbox.

Brochosomes are a peculiar kind of natural structures that hides many promises for its use in nanoplasmonics if we introduce metals into their synthetic simulacra. They are produced by small insects from the leafhoppers family (Cicadellidae) as integumental (cutaneous) powders. Structurally, they typically represent nanohole-riddled hollow spherical or ellipsoidal protein particles with dimensions typically ranging from 200 nm to 600 nm, but sometimes even extending over a few micrometers. The largest brochosomes often have elongated shape and are produced by gravid females which use them to cover and protect the laid eggs [27]. Brochosomes perform an important role of being a superhydrophobic defense against sticking to sap of plants [28]. Many systematic studies of the properties of brochosomes were published in papers by Rakitov et al. [27,28,29,30,31,32].

Yang et al. described the fabrication and properties of what they called “synthetic brochosomes”, actually diffractive nanostructured-by-templating antireflective coatings made from plasmonic materials, primarily silver [33], thus inaugurating in the field of biomimetic plasmonics the topic that makes use of the geometrical attributes of brochosome-like artificial particles. Because the advantageous electromagnetic properties of such simulacra have been swiftly recognized by the scientific community, primarily their antireflective properties in the visible spectrum, other publications dedicated to the topic of their micro/nanofabrication followed [34,35,36,37,38,39]. As far as the authors of this treatise are aware, no teams considered possible generalizations of the designs of brochosome-inspired plasmonic microparticles.

In this text, we first shortly describe plasmonic (metal-containing) analogues to the brochosomes most often met in nature. We continue by outlining a number of possible alternative designs, many of them based on existing biological structures, and by proposing a classification that could be applied to them. Based on the state of the art knowledge in nanoplasmonics, we introduce substantial generalizations and introduce novel designs, all the while remaining inspired by natural brochosomes, but at the same time fundamentally modifying the optical behavior of our proposed structures through the use of different metal-containing arrangements, since it is well known that the presence of a good electric conductor is the prime condition for the existence of plasmonic effects (i.e., there cannot be any plasmonic phenomena in the biological brochosomes, since they consist of pure dielectrics). All the structures that we describe are still either hollow or core-shell particles with surface nanostructures that may be apertures, but may also be other geometries that ensure similar functionalities, including surface protrusions/spikes and reliefs. Further, as an illustration, we perform finite element modeling of the simplest generalized plasmonic brochosome-inspired particles, with an aim to show that, even in such a case, we arrive at many novel electromagnetic properties and functionalities. We discuss our proposals and our results in some detail. We conclude that our classification can be used to extend the plasmonic design toolbox and to anticipate possible future designs. We also conclude from our numerical simulations that a very rich electromagnetic behavior of brochosome-inspired plasmonic particles is obtained even from the simplest structures, including effective optical parameters (magnetic permeability, dielectric permittivity, and refractive index) that readily reach values below 1 and even below 0 (negative refractive index metamaterials).

## 2. Materials and Methods

We analyzed ordered nanostructured metal–dielectric composites inspired by the main structural properties of natural brochosomes. We considered the use of gold as the metal of choice for this purpose. Rather than using the Drude model approximation, we utilized experimentally determined values of complex optical parameters of gold published in literature [40] to obtain more realistic modeling results. The dielectric we considered was air (refractive index *n* = 1, imaginary part equal to zero). However, most of our discussion (except the calculated numerical values) and our complete proposed classification and conceptual generalization are valid for any lossless dielectrics composited with any conductors containing free electron plasma (plasmonic material). Such conductors include, besides metals such as gold, silver, aluminum, etc., a lot of different materials, e.g., transparent conductive oxides like indium tin oxide (ITO), MXenes, graphene, etc. [41]. Solid dielectrics may be silica, aluminum oxide, magnesium fluoride, various polymers including photoresists, etc.

We designed our structures in the free 3D computer graphics software environment Blender. We simulated the electromagnetic properties of the brochosome-inspired plasmonic structures using the finite element modeling (FEM) commercial package Comsol Multiphysics, for which purpose we utilized only its RF module. The 3D models generated in Blender are readily imported in Comsol Multiphysics.

As a proof of concept, we analyzed the very simple case of a hollow gold sphere with a 600 nm diameter, its wall thickness being 20 nm, perforated by holes with a diameter of 160 nm. A pair of holes was created in the front and the back of the sphere, another pair on the left and the right side, and the third one on the top and the bottom, a total of 6 holes, each pair aligned along one of the main Cartesian axes—x, y and z. The hollow metal spheres themselves were ordered in a planar square lattice, the distance between the surfaces of each two neighboring spheres being 200 nm, both along the x and the y direction. Our reasoning was that if such a simple structure exhibited complex electromagnetic behavior as expected, then other, more complex ones should show even richer optical properties and thus extend their usability. This is a rather obvious claim—more complex systems will quite probably result in more complex behavior. The experiments presented in [33] prove this stance. Reflection measurements made with silver structures consisting of densely packed hemispherical nano-indentations on top of 2 μm microspheres showed that such structures exhibit average reflection coefficients of 1% or less across the 250 nm–2000 nm wavelength range.

To simulate the arrays by FEM, we applied periodic boundary conditions. We calculated the frequency dispersion of the scattering parameters of our structures, i.e., their coefficients of transmission and reflection. We determined the spatial distribution of the electromagnetic field intensity both in a plane passing through the centers of all the spheres in the array and on the surfaces of the spheres themselves. The array was illuminated from the top (the normal incidence). 

We also extracted the effective optical parameters for planar lattices of holey spheres. We used our simulated complex transmittance and reflectance to determine the effective relative dielectric permittivity and effective relative magnetic permeability. We further used them to calculate the effective refractive index by the method introduced by Smith et al. [42].

## 3. Results

In the first part of this section, we offer a working definition of brochosome-inspired plasmonic particles. Then, we propose a possible classification of these. We also suggest several possible novel types of brochosome-inspired plasmonic particles that satisfy the definition and could be produced by using nanofabrication techniques (e.g., self-assembly), but have not been previously described in literature. In the second part of the section, we present numerical results of the FEM simulation of an exemplary brochosome-inspired plasmonic particle, including the spectral dispersion of its reflection and absorption coefficients.

In this place, we would like to clarify an important point, namely that our consideration and actually this whole treatise regards the artificially created brochosome-inspired plasmonic particles only. No plasmonic properties have ever been demonstrated for natural brochosomes, as this would contradict the very fundamentals of electromagnetics. In other words, any brochosome-inspired structures must contain plasmonic materials (i.e., noble metals, gold in our case) in order to satisfy the basic assumptions of nanoplasmonics and general electromagnetics.

We start by defining a brochosome-inspired structure from the electromagnetic point of view as any artificial micro- or nanoscale particle that may be hollow or of core-shell type and has its surface nanostructured so that this structuring significantly modifies both its near-field (evanescent/surface-bound waves) and far-field (propagating waves) optical behavior. Such a definition is sufficiently broad to encompass all of the brochosome-inspired particles fabricated until now—e.g., [33,34,35,36,37,38,39] (which often do include plasmonic/conductive materials as one of their constituents). The definition, however, also covers a number of other structures which share similar design guidelines and exhibit a rich electromagnetic behavior, but the type and the properties of their surface nanostructuring that modifies their optical response are much wider than in those already presented until now to the scientific community. Obviously, there are whole classes of artificial structures that satisfy the given definition of brochosome-inspired plasmonic particles, but do not belong to the synthetic ones that have already been fabricated.

### 3.1. Classification

In this subsection, we propose a possible classification of brochosome-inspired plasmonic particles. It includes a number of generalizations, and albeit the list may be far from complete, it could serve as a starting point for further research.

We divide the artificially produced (or producible) brochosome-like particles into two large groups. One of them are structural (but not electromagnetic!) replicas of the biological ones, while the other one represents conceptual generalizations, mostly from the electromagnetic point of view. The latter means that, structurally, the basic core-shell design is retained, as well as most of the geometry including surface nanostructuring, but that nanostructuring differs, sometimes vastly, from that found in real brochosomes. In the further text, we delve deeper in both of the groups, describe some possible subtypes of structural generalizations, and give examples for many of them.

#### 3.1.1. Structural Replicas

An important group consists of brochosome-inspired plasmonic particles that strive to replicate the geometry and design of the natural ones as closely as the contemporary fabrication technologies allow (with an obvious difference that the artificial plasmonic structures do contain noble metals). Thus, miming of the natural designs here is on the level of the structure and geometry only. As far as the building materials are concerned, generally the choice available (and used) for the brochosome-inspired plasmonic particles is obviously wider than that for the natural ones. There is a freedom to use, e.g., numerous polymer materials for the building blocks of the brochosome-inspired plasmonic particles instead of just proteins and possibly lipids. As mentioned above, a glaring difference is that the synthetic structures often base their function on the use of metals. This imparts them numerous new functionalities compared to the natural ones, and, among other things, vastly enhances their antireflective properties due to the high light absorption in metals.

**Figure 1:**
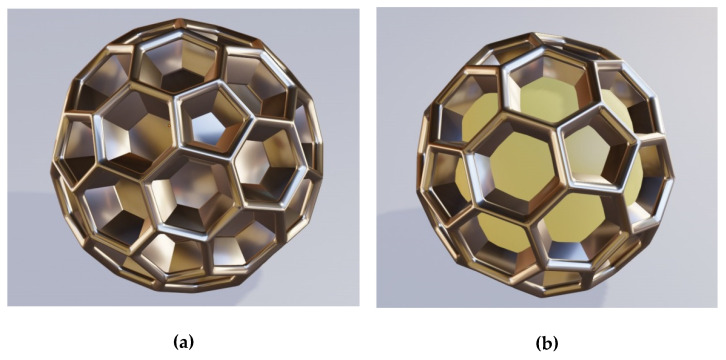
Fullerene-like honeycombed metallic structures miming the most common type of biological brochosomes. (**a**) Metal shell of buckyball-type honeycombed core shell; the core is dielectric (may be air); (**b**) Metal walls of buckyball-type honeycomb; no metal bottom is present at the bottoms of the honeycomb cells and the dielectric core is visible.

The basic type of natural brochosomes, the one that prevails by far in sheer amounts and is found in practically all Cicadellidae, takes the form of a geodesic sphere, more specifically the form of a fullerene (buckyball) upscaled to submicrometer dimensions. That is to say that its walls that are normal to the center of the round particle form a honeycomb of hexagons and pentagons. Often, a hole is located at the bottom of a separate cell of the honeycomb, leading to the air-filled core. Its diameter typically varies somewhere between 200 nm and 600 nm. Two versions of metal–dielectric replicas of this most common structure are shown in Figure 1. 

The structure in Figure 1a shows an all-metallic shell replica of the most common buckyball-shaped natural brochosome without holes at the bottom of the cells. Figure 1b shows the same type of the structure as Figure 1a, but without the metallic bottom. Only the walls are metallic in this case, while the interior (visible) is filled with solid lossless dielectric.

It is well known fact in plasmonics that all the usual, propagating electromagnetic waves (which also include visible light) have their wave vectors much smaller than the evanescent (exponentially decaying with the distance) surface plasmon polariton waves that are bound to the interfaces between metal and dielectric. Since the presence of evanescent surface waves represents a mandatory condition for the existence of plasmonic effects, it is always necessary to perform matching between the propagating and the evanescent waves. This is done by imparting additional wave vector to propagating waves if we want our plasmonic structure to be functional at all. 

In the case of metal-containing brochosome-inspired microstructures, this electromagnetic coupling of the incident propagating light waves with the evanescent surface waves proceeds by electromagnetic diffraction. This interaction imparts the mentioned necessary additional wave vector to the incident optical wave, as required for its coupling with the surface plasmon polariton. Electromagnetic waves enter the interior of the brochosome-like structure in a fashion similar to that in the well-known extraordinary optical transmission structures where light propagates through apertures with subwavelength dimensions (in spite of the fact that according to the conventional electromagnetic models there should not be any transmission at all). The details on how this electromagnetic process actually takes place can be found in, e.g., [43,44]. 

A curiosity is that a nanohole does not even have to be cut throughout the sphere shell, perforating its inner surface in order to allow the transmission of waves through it. From the plasmonic point of view, it is sufficient that the hole goes only partly into the shell, leaving a pit of a finite depth and creating surface corrugation that will result in electromagnetic diffraction. Even when this is the case, the evanescent field will couple with the opposite (inner) side of the shell surface and form an evanescent field within the dielectric interior of the structure nevertheless. The electromagnetic mechanism of this coupling is described in, e.g., [45] and exceeds the scope of this paper. 

A problem with the type of metal simulacrum shown in Figure 1 is that it may be a rather challenging task to nanofabricate them. It appears that the safest approach could be to just use natural structures for templating and to deposit a layer of metal over them by, e.g., radio-frequent sputtering in a manner similar to the one described in [14]. 

Another important type of natural brochosomes is the “cratered” one where the surface is covered by densely packed hemispherical pits. This type of brochosome is found in the leafhopper species Oncometopia alpha (as shown in Figure 7Q of the paper by Rakitov [30]). Its diameter is relatively large and is of the order of 1–2 micrometers or even more. The experimentally produced particles loosely inspired by biological brochosomes, as reported by Yang et al. in 2017 in *Nature Communications*, resemble somewhat this type [33]. However, Yang’s replicas of cratered brochosomes were fabricated by templating only the top part of the surface with sacrificial nanospheres, as the main goal of that research team was to maximize antireflective effects from the top side.

**Figure 2:**
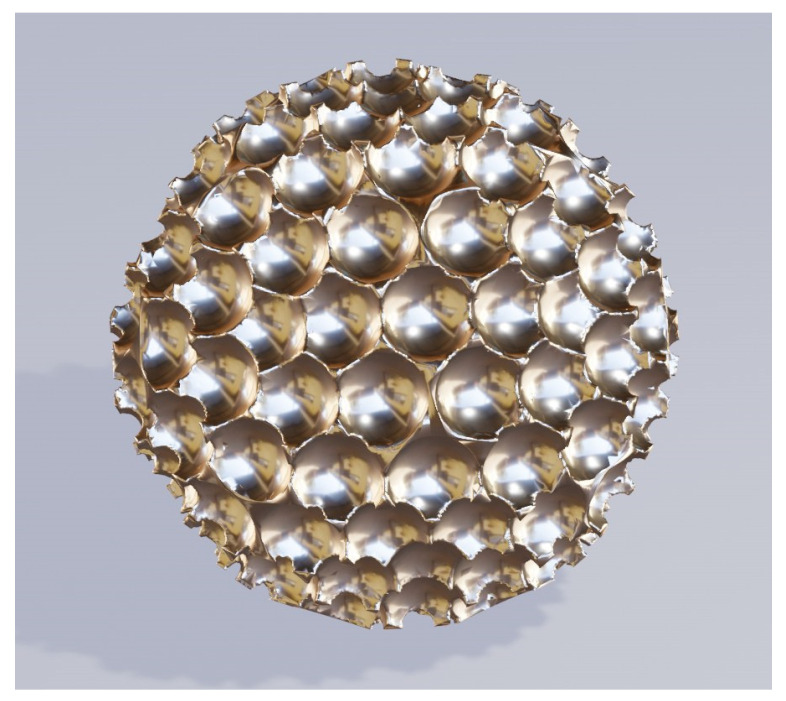
Metal replica of the Oncometopia cratered brochosome-like shell.

Figure 2 shows a metal shell replica of the Oncometopia alpha cratered brochosome [30]. In our case, the coverage with hemispherical pits is extended over the whole surface. The core of the pitted sphere is dielectric (not to be seen in the figure), while the external shell is gold.

#### 3.1.2. Conceptual Generalizations

In order to be consistent with the natural brochosome geometries and for the sake of simplicity, here, we consider only spherical or roughly spherical overall shapes of brochosome-like plasmonic particles. In reality, many other 3D shapes could be built in the core-shell configuration and with surface nano-features like holes. Besides spheres, these include ellipsoids, various polyhedrons including stellated (nonconvex) ones, prisms, antiprisms, pyramids, bipyramids, trapezohedrons, toruses, Dupin cyclides, egg-shaped and lemon-shaped forms, truncated closed hyperboloids and paraboloids, and many more. Our present consideration can be straightforwardly extended from spheres to any of the listed 3D shapes.

**Figure 3:**
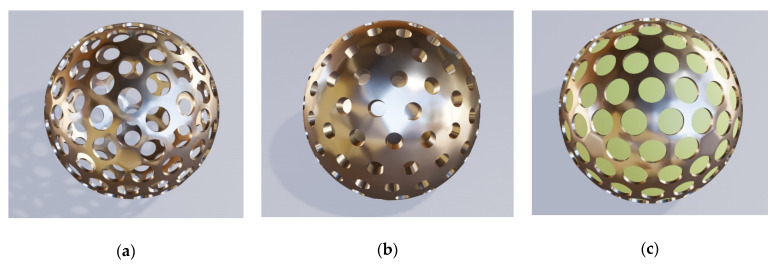
(**a**) Hollow sphere with uniform and regularly patterned holes; (**b**) the same hollow structure, but with a smaller hole diameter and with a thicker metal shell; (**c**) the same structure as in (**a**), but with a core of solid dielectric.

We start by considering the simplest of the forms, a regular sphere with its shell riddled with cylindrical holes and with a core that is either hollow or filled with material different from that of the shell (primarily lossless dielectric). Figure 3 shows three different but similar cases of golden core-shell spheres with cylindrical nanoholes. Figure 3a shows a hollow sphere with uniformly distributed and regularly patterned holes with larger diameters, Figure 3b presents a very similar hollow structure, but with smaller hole diameter and with a thicker shell, while Figure 3c shows a sphere with its dimensions equal to those shown in Figure 3a, but with a core of solid dielectric. All three illustrations obviously represent variations on the same theme.

The spatial distribution pattern of the nanoholes shown in Figure 3 is ordered—the apertures are arranged in a lattice of equilateral triangles. The nanoholes can be also ordered in a square lattice or a hexagonal one. The ordering may be more complex—for instance, the lattice constant may be graded along one or two directions or the nanoholes may be grouped according to some rule. For instance, they may follow a quasiperiodic or aperiodic layout. Obviously, they might be fully disordered, assuming random positions on the sphere surface.

As far as the shapes of the apertures are concerned, they can have any imaginable form: cylindrical, hemispherical, prismatic with any form of the prism base, including various stellate shapes, crosses, C- and S shapes, and actually any arbitrary regular or irregular form. The walls of an aperture do not have to be parallel—openings can be cone-shaped, pyramidal, hemispherical, etc. Let us remember here that a nanohole does not have to penetrate through the inner surface of the shell in order to ensure evanescent wave transmission to the inner surface, owing to the diffractive coupling with the illuminating wave mediated by surface corrugation [45].

Regarding the nanohole shapes in plasmonics, the sharper their edges are on a deep subwavelength scale, the higher electromagnetic field localization will be on them (the edge effect). The same is valid for the nano objects in close vicinity to each other (the proximity effect). In the final instance, this leads to the near field hotspots and to vast concentrations of fields. Consequently, it results in a breakdown of the effective medium approximation which, in its usual form, does not account for the hotspots. More details can be found in, e.g., [46,47,48].

**Figure 4:**
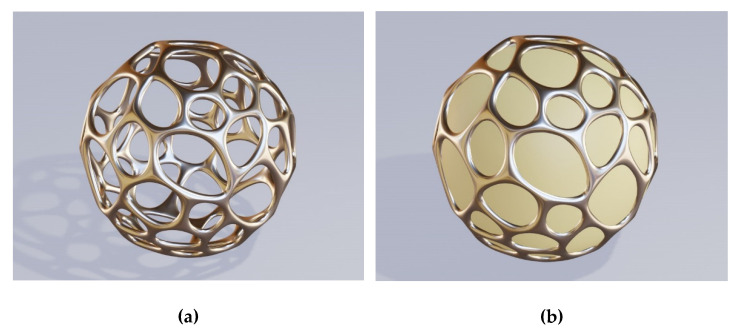
Holes designed to follow Voronoi pattern; (**a**) the core of the sphere with Voronoi openings is hollow; (**b**) the core is a solid lossless dielectric sphere.

The hole shapes may vary across the surface according to some pattern which may be more or less complex. Figure 4 shows an example of one of the more complex dependencies where the holes are shaped as the Voronoi pattern (all points of a hole are built around a seed point in such a manner that all points defining a hole are those nearest to it [49]. Metal parts are at the edges of Voronoi areas. Figure 4a shows the case where the core of the sphere with Voronoi openings is hollow, while Figure 4b represents the case where the core is a solid lossless dielectric sphere.

Finally, the nanohole shapes may vary randomly. In this case, the perforations will be completely irregular. Here, one encounters again the edge effects and the proximity effects that additionally enhance the field concentration.

A similar set of rules valid for the hole shape classification is applicable to the shape size. The hole size may be constant across the whole sphere or only across a part of it. Alternatively, the hole size may vary around the sphere following a more or less complex set of rules. Actually, Figure 4 shows holes with both their sizes and shapes changing in the form of the Voronoi pattern. The number of rules that may be followed by the hole sizes is literally endless. Of course, one can also fabricate nanoholes without any given set of rules, and the hole size will randomly vary across the brochosome-inspired plasmonic microparticle.

The geometry of the brochosome-inspired plasmonic microparticles can be inverted, so that where one encounters holes in the original structures, there will be protrusions or surface nanoparticles. In plasmonics, according to Babinet’s principle [50], the diffraction patterns, including deep subwavelength near field hotspots, remain identical if an aperture is replaced by a particle/protrusion with the same geometry. A difference is that the in-plane electric components of the near electromagnetic field become mutually interchanged with their corresponding magnetic components with their polarization perpendicular to the electric ones. For this reason, one usually also interchanges the dielectric and the metallic parts of the structure. Thus the behavior of a brochosome-inspired plasmonic microparticle with surface nano-protrusions will remain quite similar to that of a holey structure, except obviously in the cases when the geometries do not fully coincide, as is the case with e.g., surface nanowires. Based on Babinet’s principle, surface indentations and protrusions can be regarded as equivalent in mathematical, electromagnetic, and functional sense.

Skipping from the nano-indentations (as met in biological brochosomes) to nano-protrusions in brochosome-inspired plasmonic particles may at a first glance appear counterintuitive, illogical, and even plain wrong since, from the geometrical point of view, they look as if they do not bear a semblance to each other. From the electromagnetic and functional point of view, however, the structures with indentations and those with protrusions are interchangeable, as described above. Moreover, we argue below that they both share the same inspiration by the biological brochosomes since the only modification that we introduced is actually the placement of protrusions in the location of holes, as justified by Babinet’s principle. Thus, such extension from perforations to protrusions represents a logical extension of the geometry of the brochosome-inspired plasmonic particles. In other words, if we investigate microparticles with their surfaces adorned by nanostructuring, then from the electromagnetic point of view, it is unimportant if these adornments extend towards the microparticle interior or towards the outside: both share the same electromagnetic concept. They represent two faces of the same picture and this treatise would be sorely lacking without any of them. This unification of indentations/perforations and protrusions/spikes is one of the major points of the conceptual generalization introduced in this manuscript. The chosen approach vastly enriches the design possibilities and the range of available plasmonic properties.

While holes can be fabricated by subtractive nano-processing (physical or chemical etching) of the metal shells, protrusions can be produced by additive approach (material deposition or growth), possibly combined with etching. For the fabrication of both types of nano-features (holes and protrusions), self-assembly techniques can be applied for templating. In the case of protrusions, these techniques can be also used for direct growth of the surface features.

Since the protrusions represent an inverse geometry with regard to holes, similar considerations are valid for their classification. Thus, the protrusions can also have the form of regular 3D geometrical objects, including cylinders with circular or elliptical basis, various polyhedrons, pyramids, and prisms with a variety of bases including regular polygons, but also irregular ones (I-shapes, L-shapes, T-shapes, crosses, etc.), cones, hemispheres, nanowires, etc. The surface protrusions can also have any irregular shape, the main factor here being the mechanical stability of the structure.

As far as the protrusions sizes are concerned, similar rules are applicable as for the holes, with one crucial difference: the longitudinal dimensions of protrusions may vastly exceed those of the holes and may indeed exceed the diameter of the main sphere itself. An example would be a 2D array of self-assembled nanowires on the surface. The size of various protrusions can be constant across the sphere, may vary in both transverse and lateral direction according to a more or less complex set of rules or irregularly (randomly).

**Figure 5:**
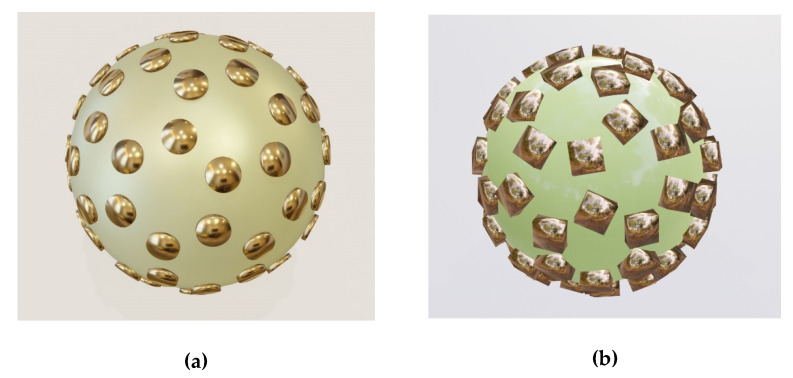
Brochosome-inspired plasmonic spheres made of dielectric with metallic surface nanoparticles corresponding to the holes in metal shell. (**a**) Low-height cylinders (disks) distributed across the surface in the form of a hexagonal lattice; (**b**) flattened square cuboids distributed across the surface in the form of a hexagonal lattice.

Figure 5a shows the Babinet’s structure inverse to the one shown in Figure 3. Here, we have cylinder or disk-shaped surface nanoparticles distributed evenly across the surface in a hexagonal lattice. In this case, the base material is solid lossless dielectric, while the protrusions are metallic. The electromagnetic behavior of the structure remains quite similar to the holey case represented in Figure 3.

As a variant of the structure in Figure 5a, in Figure 5b, we show an array of regularly distributed rectangular shapes in the form of square cuboids. It could be expected that their electromagnetic response should include a highly increased number of field hotspots due to a large number of sharp edges.

**Figure 6:**
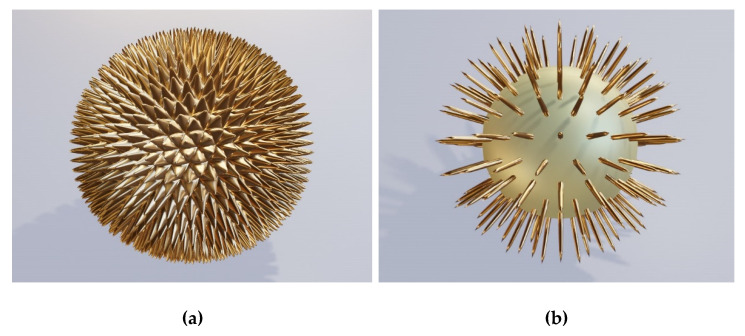
Brochosome-inspired plasmonic spheres with metal spikes on the surface; (**a**) rounded trigonal pyramids; (**b**) Surface metal wire-like spikes.

Extending the concept still more in the same direction, we arrive at structures adorned pyramidal spikes (Figure 6a) and with nano-spikes (Figure 6b) which make use of the edge effect even more. Mind that we still follow the same basic concept, but extend it further.

The geometries shown in Figure 6 actually appear like scaled-down versions of geometries of some species of Radiolaria [51,52] (whose typical diameters are a few hundred micrometers)—albeit, in this case, much smaller and made at least partly from noble metal. We believe that these may be the first proposed designs of biomimetic plasmonic structures inspired by Radiolaria.

**Figure 7:**
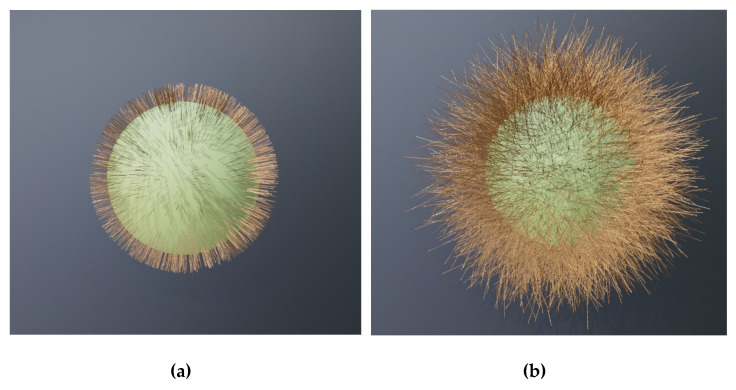
Brochosome-inspired microspheres with metal nanowires grown on the dielectric shell surface; (**a**) vertically grown cylindrical metal nanopillars; (**b**) disordered metal nanowires.

Figure 7 shows other kinds of surface protrusion-based brochosome-inspired plasmonic particles, the structures based on self-assembled nanowires [53,54,55] grown on lossless dielectric spheres with submicrometer diameters. At a first glance, these structure look as if they do not have anything in common with brochosome-like particles, but we just showed that they actually represent their logical extension. Figure 7a shows ordered cylindrical metallic nanopillars [55] of approximately constant heights and diameters, while 7b shows disordered metal nanowires with varying lengths and growth directions.

The next large group of brochosome-inspired plasmonic particles includes spherical structures with nano-reliefs—i.e., spheres with sculpted shells. These particles again share some geometrical traits with natural brochosomes, but generalize them in the sense that now, we have grooves instead of separate holes/indentations. Actually, the sculpting may be either subtractive (grooves) or additive (ridges). Thus, with this type of structuring we extend our concept even further.

This group with nano-reliefs can be further divided into several subgroups. Among them are spheres with grooves or ridges of simple regular shape. An illustration of this type of relief would be particles with sets of parallel grooves/ridges, miming the look of standard diffractive gratings on a subwavelength level. The lines defining these grooves/ridges may be straight or curved. Another illustration consists of intersecting lines, which again may be straight or curved.

Another subgroup is made of complex nanopatterns obeying a more or less complex mathematical rule. Among examples of such reliefs are those based on Turing patterns, as described in his seminal paper “The chemical basis of morphogenesis” [56]. This is a form ubiquitous in nature, from nano-world to macro-world. It is based on the diffusion reaction, occurring for instance during self-assembly of nanostructures based on block-copolymers, an approach often used for nanopatterning and nanolithography [57,58].

**Figure 8:**
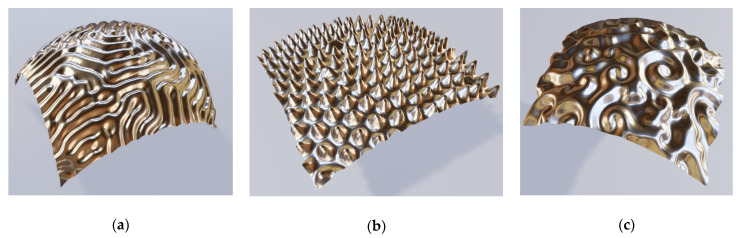
Calottes with templated reliefs corresponding to Turing patterns of diffusion; (**a**) diffusion reaction lines; (**b**) diffusion reaction dots; (**c**) complex Turing diffusion reaction pattern.

Three examples of the nanostructured spherical surfaces are shown in Figure 8. We calculated all of them using the diffusion reaction formulas with various parameters. The leftmost figure (a) shows a pattern of curvilinear surface profiles. By changing the parameters in the differential equation defining the Turing pattern, we obtained a dotted distribution where the dot positions follow the Turing rule (b). A more complex profile, also defined by the diffusion process, is shown in (c).

**Figure 9:**
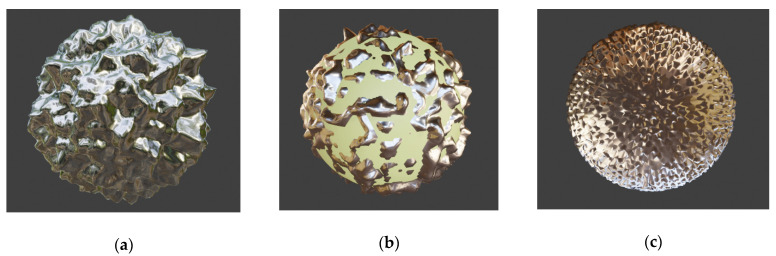
Irregular (stochastic) nano-reliefs of the sculpted shells of brochosome-inspired plasmonic particles. (**a**) Randomly sculpted continuous silver shell; (**b**) corrugated metal islands on a dielectric sphere; (**c**) Randomly scattered surface protrusions on a continuous golden shell.

The surface reliefs can also be completely irregular (stochastic). A few typical examples are shown in Figure 9, including continuous metal shells with random reliefs and corrugated metal islands on a dielectric core (a Babinet’s equivalent to a holey structure with randomly sculpted metal shell). Obviously, the number of possible stochastic reliefs is literally endless. We note here that both Figure 8 and Figure 9 actually represent combinations of surface indentations and protrusions.

**Figure 10:**
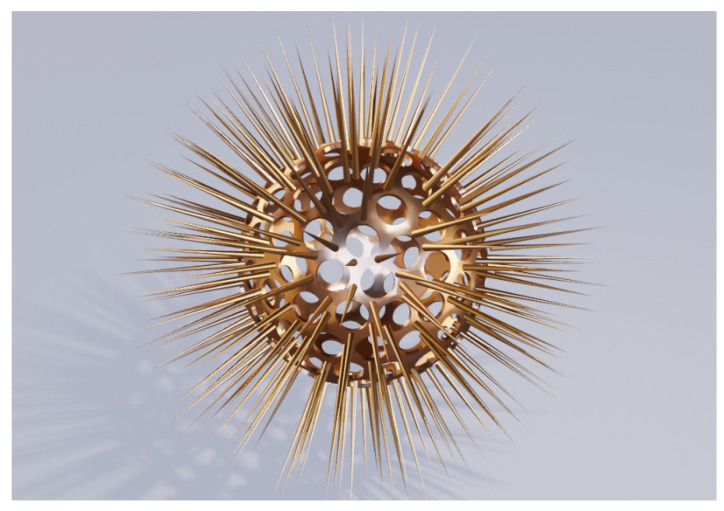
Combined perforated and spiked hollow metal structure.

Finally, a brochosome-inspired plasmonic particle may represent any combinations of two or more above presented structures. An example could be a particle with its surface adorned with nanoscale apertures and spikes at the same time. A possible structure is presented in Figure 10. We intentionally chose to show as an illustration a particle with geometrical features of Radiolaria, scaled down to nano-dimensions and with plasmonic properties. We see that however we may strive for a generality of our approach, nature will remain the prime source of inexhaustible inspiration.

In Table 1, we present a summative review of the brochosome-inspired plasmonic particles described in the previous text. We summarize the possible structures and their proposed classification. It can be seen that many of the generalizations quoted in Table 1 and described in the division 3.1.2. (Conceptual Generalization) that appear in biological forms are represented here (the ubiquitous Turing patterns—the result of diffusion reaction—but also Voronoi forms, Radiolaria geometries, etc.).

### 3.2. Simulations

We performed our calculations of optical properties of a simple spherical brochosome-inspired plasmonic particle with six nanoholes by the method described in Section 2. The approach that we applied to determine the complex transmittance and reflectance, as well as complex values of the effective relative magnetic permeability, effective relative dielectric permittivity, and effective refractive index, is described in more detail in [59]. Here, we present some of the results and comment on them.

**Figure 11:**
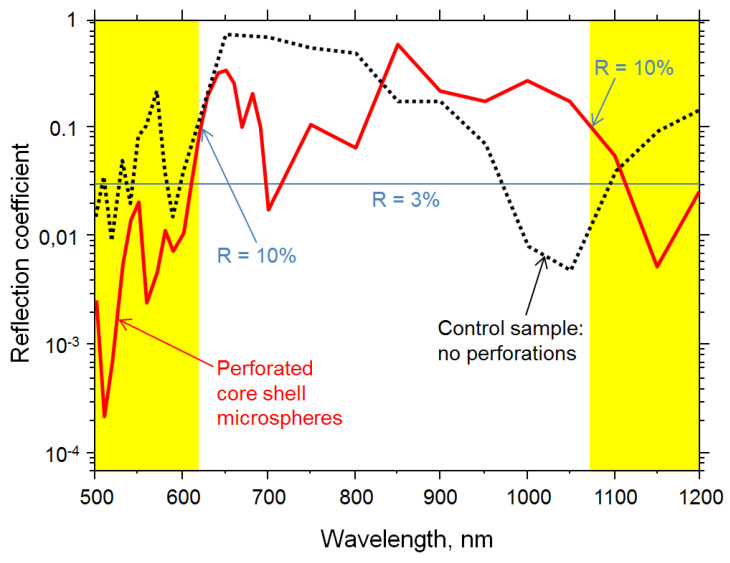
Solid red line: spectral reflection coefficient of a square planar array of golden hollow spheres with a 600 nm diameter, a wall thickness of 20 nm, perforated by holes with a 160 nm diameter and with a distance between two neighboring spheres equal to 200 nm. Dotted black line: the same dependence for a control sample, having the identical geometry and materials, but without perforations. Yellow fields at short wavelengths (500 nm—620 nm) and at long wavelengths (1070 nm—1200 nm) denote the spectral ranges where the reflection coefficient does not exceed 10%, while the blue horizontal line is the 3% limit of the reflection coefficient. The superior antireflective properties of perforated core shells at shorter wavelengths are clearly observable.

**Figure 12:**
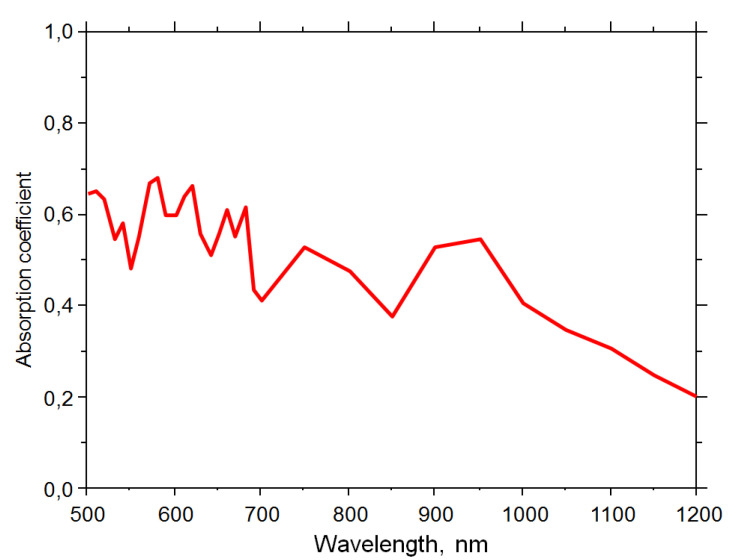
Spectral absorption coefficient of a square planar array of gold hollow spheres with a 600 nm diameter, their wall thickness 20 nm, perforated by holes with a 160 nm diameter and a distance between the surfaces of two neighboring spheres of 200 nm.

The calculated optical parameters are depicted in the spectral diagrams shown in Figure 11 and Figure 12. For the sake of clarity, we chose to show here only the reflection (Figure 11) and absorption (Figure 12) coefficients, the parameters of interest for antireflective behavior that has been the prime target of several publications that inaugurated the field of brochosome-inspired microparticles. If necessary, the transmission coefficient can be readily calculated by applying the simple and well known relation *T* = 1 − *A* − *R*.

In order to ensure a better insight into the antireflective behavior of the simple brochosome-inspired plasmonic microspheres, we present the spectral dependence of their reflection coefficient in Figure 11 in logarithmic scale. The lowest values of the reflection coefficient are seen at the shortest wavelengths considered here, between 500 nm and 615 nm (green-yellow color), where the dimensions of apertures (160 nm diameter) are clearly subwavelength. In most of this range, the reflection coefficient does not exceed 3%, its value at some wavelengths reaching down to almost 0.01%, and not increasing above 10%. The next range of low reflection coefficient is observed at wavelengths between 1070 nm and 1200 nm (near-infrared), where the maximum reflection coefficient again does not exceed 3%.

In order to compare the results to the case without perforations, we did the same calculation for an identical setup (both geometry- and material-wise), but with solid gold spheres. It can be seen that brochosome-inspired microparticles exhibit superior antireflective behavior at shorter wavelengths (between 500 nm and 600 nm), i.e., in the range where the resonances are limited to the microspheres themselves. As expected, we observed that at those short wavelengths the lowest reflection coefficients of the perforated spheres are one to two orders of magnitude lower compared to the unperforated case, just the point we intended to prove. As one could expect, the resonances at near-infrared wavelengths, which are determined by the far-field effective optical behavior of the whole array, are comparable between the two cases, the only significant difference being in different spectral shifts of their minima. This all is a consequence of material behaving as an effective medium at longer wavelengths—the interrogating light beam is unable to “see” the nano-perforations.

Another important difference between the brochosome-like case and the unperforated gold spheres is that we did not observe metamaterial effects in the latter. Thus, the control sample exhibits much less rich and desirable plasmonic behavior than the brochosome-inspired one.

Figure 12 shows the absorption coefficient of the simple brochosome-inspired plasmonic particles in the range of 500 nm–1200 nm in linear scale. Throughout this range, a trend of decrease of the absorption coefficient towards longer wavelengths is clearly visible.

**Figure 13:**
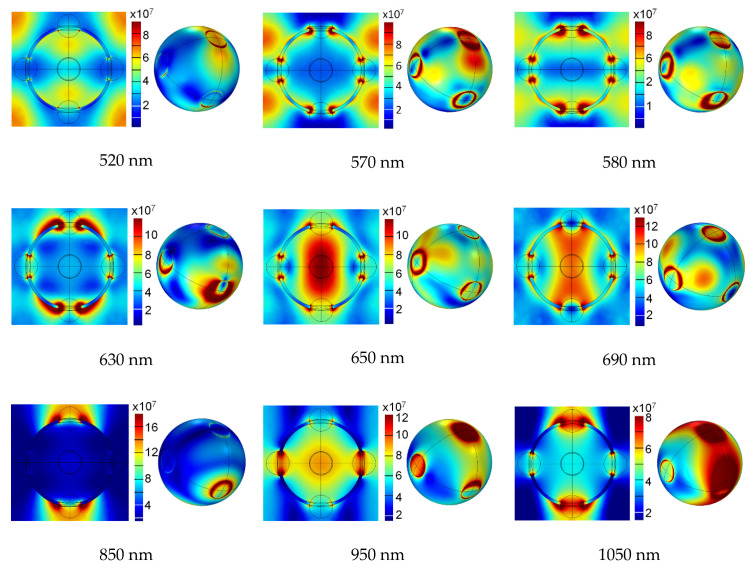
Spatial distribution of electric field in a unit cell of planar square lattice of simple brochosome-inspired plasmonic particles (**left part of each sub-picture**) and the field on the surface of a single brochosome-inspired particle (**right part**). The design data are the same as in Figure 11 and Figure 12. The operating wavelengths for each case are given below the corresponding sub-picture.

The spatial distribution of the electric component of the optical field was calculated at various wavelengths both for a single unit cell of the planar square array of perforated core-shell gold-air particles (showing the distribution at the mid-particle cross-section and in the space between the particles) and at the surface of a single plasmonic holey sphere. Figure 13 illustrates the variety and richness of electromagnetic effects at this seemingly very simple structure.

The electric field distribution at the cross-section of a unit cell including both the holey sphere and the inter-particle space is shown in the leftmost part of each sub-picture, while the field distribution across the surface of a single holey sphere is shown in the right. The design data including materials and full geometry is the same as in Figure 11 and Figure 12. The operating wavelengths for each case are given under the corresponding sub-picture.

It is noticeable from Figure 13 that the electromagnetic behavior of the holey particle array drastically changes with the operating wavelength. Some peculiarities are visible at a first glance. However, the overarching principle is completely consistent and analogue with the case when nanoapertures are opened in a planar metal sheet. Light scatters on the edges and the apertures (for instance, dipole modes can be readily seen around the nanoholes) and these scattered near field components couple with each other (both intra-particle and inter-particle coupling). This coupling can be constructive or destructive and forms a singular optical response of the structure. As such, this response on a single particle level can vary from circularly distributed individual scatterers to that of a full metallic shell with tailored effective parameters and everything in between.

At shorter wavelengths (500 nm to 600 nm), the field also couples with the surrounding spheres, creating leaky modes. Thus, the field coupling at these short wavelengths is weak, and absorption at the spheres is high since localized surface plasmon polaritons are strongly absorbed in the metal shell both at its outer surface and on its inner surface. This is also visible from the absorption dependence in Figure 12. Overall, this creates a strong antireflective behavior and a large drop of the reflection coefficient, which, as mentioned, goes down below 0.02% at certain short wavelengths. Narrow regions of highly concentrated fields at the edges of nanoholes can be seen at these wavelengths.

At longer wavelengths, we observe very rich spectral electromagnetic behavior. The response of the holey spheres shows strongly reflective nature at 850 nm, where the field coupled with the insides of the sphere is very weak. Contrary to that, at other wavelengths the inner near field intensity is very high, as seen at e.g., 650 nm and 690 nm. This contradicts the usual macroscopic far field behavior where the electric field would be almost nonexistent inside of large metallic spheres. The field distribution at the surface of the spheres is in many cases reduced to annular hotspots near the nanoholes’ edges, but at longer wavelengths, it is quite high.

**Figure 14:**
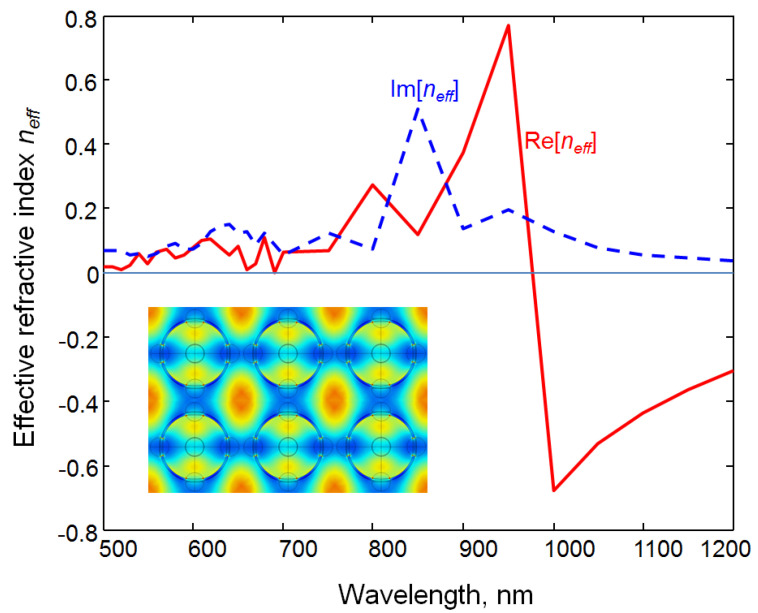
Spectral dependence of extracted effective refractive index of a square planar array of gold hollow spheres with a 600 nm diameter, their wall thickness 20 nm, perforated by holes with a 160 nm diameter and a distance between two neighboring spheres 200 nm. Solid red line: real part of the effective refractive index of the array of brochosome-like perforated microspheres. Dashed blue line: imaginary part of the effective refractive index of the array of brochosome-like perforated microspheres. Inset: 6 unit cells of the square array of brochosome-like perforated microspheres showing electric field distribution at 520 nm.

We also extracted the effective refractive index for a planar array of holey spheres with square lattice. We followed the approach from [42,60], and details about the calculation of effective permittivity and permeability for this particular type of particles can be found in our own text [59]. The real and the imaginary part of the calculated effective refractive index are shown in Figure 14.

Similar to the results previously described for brochosome-inspired microparticles, Figure 14 shows a rich electromagnetic behavior. The peculiarities observed include the metamaterial behavior of the array, with refractive index values lower than 1, even reaching near zero at some wavelengths, and negative at longer wavelengths (double negative metamaterial).

## 4. Discussion and Conclusions

Our results dedicated to classification (Section 3.1) show that a large diversity of shapes and forms of brochosome-inspired particles can be achieved by introducing into their designs some kinds of biomimetic structuring seen in other types of organisms besides brochosomes. We utilized natural structural solutions found in other organisms (Radiolaria, for instance), but also more general biological laws that can be met in numerous organisms (Turing patterns, met in a tremendous number of organisms, from the simplest to the most complex ones—the tiger and leopard fur patterns are determined by the same set of rules that determines the nano-world.) Thus, building blocks inspired by one type of biological structures can be used as subunits for the fabrication of final structures that are inspired by quite different types of living organisms.

From the point of view of plasmonics and metamaterials, we made a systematization of some of the existing planar patterns, including, e.g., extraordinary optical transmission holes and indentations, apertures and protrusions arrays known from theory of metasurfaces, etc., and wrapped them around a sphere. It is peculiar that we encountered very similar nanostructures and patterns in both plasmonics and biomimetics, albeit they are functionally as far from each other as possible and appear completely unconnected.

In this treatise, we did not dwell long on the role of the choice of materials, in spite of its obvious importance (there are no plasmonic effects without good electron conductors.) This was done on purpose, in order to retain a maximum generality of our approach. Besides that, this topic belongs to the conventional material science and represents a completely different field of investigation (the geometrical structures and the electromagnetic properties being our main topics here) and can be treated separately. We opted instead to consider only the structures obtainable by the standard micro and nanofabrication materials and techniques that are routinely used in MEMS (microelectromechanical systems) and NEMS (nanoelectromechanical systems) and solely the materials commonly applied in them. For instance, we chose gold as the plasmonic conductor, since it is probably the most frequently used conductor in MEMS and NEMS. Even the block copolymer self-assembly for nanopatterning has been adopted for the MEMS/NEMS a relatively long time ago.

Regarding our calculation results (Section 3.2, Simulations), for the sake of clarity, we included a part of our discussion in the mentioned Subsection, since we believed it was more convenient for the readers to see a particular discussion near the figure it analyzes. Thus, we opted to present in this section only a general discussion of the calculation results.

Our analysis of the simplest brochosome-inspired particles showed that they exhibit the largest antireflective properties at the shortest wavelengths, between 500 nm and 600 nm, their reflection coefficient being the lowest in that range. The next range of interest are the near infrared wavelengths (1070 nm to 1200 nm) where the dimensions of holey spheres become smaller in comparison to the operating wavelength, so that their planar array begins to behave as an effective medium. The conventional effective medium approximation, however, will cease to be valid (e.g., [61]), because the surface nanostructuring will behave as a rich source of hotspots of highly increased field. Therefore, the applied effective approximation will have to take the nonlocal effects into account [62]. Our simulated structure will behave as a sophisticated version of artificial dielectric [63,64] in which metal particles are replaced by metamaterial particles. The effective refractive index in that range will be negative, and both absorption and reflection coefficients will be low.

Another segment that we would like to stress is that the optical behavior of the simulated holey spheres varies strongly across the spectrum. It indicates a wealth of different wavelength-dependent optical properties in a single structure, which points out to a potential for versatile practical applications.

As far as possible practical applications are concerned, the brochosome-inspired particles produced until now, although being in their infancy and belonging to quite a narrow range of designs, found their applications in such diverse areas as omnidirectional antireflective structures [33,38,39], electrochromic structures and materials [65], photoanodes for photoelectrochemistry [66], SERS (surface-enhanced Raman scattering) [36], superoleophilic and superhydrophobic submicrometer particles [34], etc. Thus, it stands to reason to expect that a vast extension of the range of possible structures might imply a proportional extension of the applicative potential. If nothing, it would introduce many existing advantages of metamaterials and plasmonic crystals to generalized brochosome-inspired particles as handled in this text and impart at least a fraction of their usability to practical applications.

Following the analogy with their planar analogues, we dare envision the use of generalized brochosome-inspired particles in chemical and biological sensing, photocatalysis and electrochemistry including novel energy harvesting devices, fuel cells, hydrogen production, photodetectors with enhanced performance, novel optical, and plasmonic passive and active elements including a novel generation of displays and many more. Shortly, we expect that the existing variety of the properties and uses of planar metasurfaces and plasmonic crystals could be imparted to submicrometer particles with nano-functionalized surfaces, since they share a lot of common functional and electromagnetic traits. Of course, there is still an enormous path to be crossed in the future, especially with regard to the particular experimental investigations.

The general message of this treatise is that by systematically cataloguing possible forms of brochosome-inspired plasmonic microstructures with nanostructured surfaces producible by the state-of-the-art nanofabrication technologies and by giving some concrete illustrations, we strived to establish a source of ideas and directions for potential future research. Our mentioning of the nanofabrication techniques that could be used to produce at least some of the described structures goes in the same direction. Shortly, we intended to lay a foundation for at least one little branch of a roadmap of possible research in biomimetic submicrometer plasmonic particles with engineered surfaces.

## Figures and Tables

**Table 1:** A proposal of a classification of the potential generalizations of brochosome-inspired plasmonic particles.

Surface Features	Variations of Surface Features	Comment
Spatial layout of apertures and protrusions	Ordered (lattice—periodic pattern) Complex ordering (e.g., quasiperiodic, aperiodic) Disordered (Random)	Distribution of surface features (holes, cylinders, nanowires, etc.)
Apertures: shapes	Constant hole shape across sphere (e.g., cylindrical, prismatic including stellate, or actually any shape of the base, hemispherical, cross-shaped, C and S-shaped, cone-shaped, pyramidal, but also irregular) Varying hole shape (defined by more or less complex rules—e.g., Voronoy pattern) Varying hole shape (random)	Subtractive processing of shell surface (perforation)
Apertures: sizes	Constant hole size across the sphere Varying hole size across the sphere (the distribution defined by a more or less complex set of rules) Randomly varying hole size across the sphere	Subtractive processing of shell surface (perforation)
Protrusions: shapes	Regularly shaped nano-bodies: cylinders, polyhedrons, stellated polyhedrons, cones, pyramids, hemispheres, crosses, split ring resonators, nanowires, etc. Irregular shapes	Additive processing of shell surface (Babinet inverse of the surface holes)
Protrusions: sizes	Protrusions (nanoparticles) with constant size across the sphere Protrusions with varying size across the sphere (defined by more or less complex set of rules) Randomly varying protrusion size	Additive processing of shell surface (Babinet inverse of the surface holes)
Surface reliefs	Regular forms (diffractive gratings—linear or curvilinear, crossed lines, 1D arrays of any of the form listed under protrusion shapes, reliefs that can be described by any type of mathematical equation) Complex regular form (e.g., Turing patterns), describable e.g., by a differential equation or a set of differential equations Irregular (random) reliefs	Additive or subtractive processing of shell surface in order to obtain variations of the heights of its various points (different distances from the center of the sphere)
Combined forms	Any combination of two or more of the above	Superposition of subtractive and/or additive forms
